# Antenatal Corticosteroids and Their Effects on Maternal Glycemic Status: A Prospective Observational Study From an Indian Tertiary Referral Center

**DOI:** 10.7759/cureus.60043

**Published:** 2024-05-10

**Authors:** Anil Satyaraddi, Basavaraj G Sooragonda, Akkamma A Satyaraddi, Kranti Khadilkar, Shivaprasad KS, Lavanya Kiran, Subramanian Kannan

**Affiliations:** 1 Department of Endocrinology, Diabetes and Metabolism, S. Nijalingappa Medical College and Hanagal Shree Kumareshwar (H.S.K) Hospital & Research Centre, Bagalkot, IND; 2 Department of Endocrinology, Diabetes and Metabolism, Narayana Hrudayalaya, Bengaluru, IND; 3 Department of Obstetrics and Gynaecology, S. Nijalingappa Medical College and Hanagal Shree Kumareshwar (H.S.K) Hospital & Research Centre, Bagalkot, IND; 4 Department of Obstetrics and Gynaecology, Cloud Nine Hospital, Bengaluru, IND

**Keywords:** gestational diabetes mellitus (gdm), persistent hyperglycemia, diabetes in pregnancy, betamethasone, hyperglycemia in pregnancy, antenatal steroids

## Abstract

Background

Antenatal corticosteroids prevent multiple fetal complications and improve overall neonatal survival but at the cost of adverse effects including maternal hyperglycemia. This study aimed to understand the effect of antenatal corticosteroids on maternal glycemic control.

Methodology

This prospective observational study included 93 pregnant women with singleton pregnancies between 32 and 37 weeks gestation admitted for potential preterm labor. We assessed their glucose tolerance and categorized 56 participants with normal glucose tolerance in group 1, while 37 who had diabetes mellitus (DM) were categorized in group 2. Of the women with DM, 30 had gestational diabetes mellitus and seven had pre-existing type 2 diabetes. Betamethasone was administered as per the standard of care, two doses of 12 mg each, 24 hours apart. To assess the effect of corticosteroids on maternal blood glucose control, we monitored capillary blood glucose levels at specific time intervals for three days following the steroid administration. Fasting and post-meal glucose levels were checked a week after the administration of the steroid therapy, and it was observed that participants from group 1 had developed steroid-related hyperglycemia. Blood glucose levels ≥140 mg/dL were considered significant hyperglycemia, while blood glucose levels ≥160 mg/dL were considered severe hyperglycemia. Following this observation, we documented any modifications in the diabetes management plan during or after the corticosteroid treatment, including medical nutrition therapy, addition of oral anti-diabetic medications, commencement of insulin, or increasing insulin dosage. Standard software programs such as Microsoft Excel and SPSS (IBM Corp., Armonk, NY, USA) were used to analyze the collected data, summarize the findings, and identify any statistically significant relationships between the variables descriptive and inferential statistics, respectively.

Results

Participants from both groups demonstrated worsening glycemia requiring treatment involving insulin, following corticosteroid administration. The percentages of significant hyperglycemic participants from groups 1 and 2 were 72% and 92%, respectively. Severe hyperglycemia was seen in 43% and 84% of the participants from groups 1 and 2, respectively. An intervention involving insulin administration was required by group 2 participants with pre-existing diabetes within six hours of steroid administration, followed by those with gestational diabetes requiring intervention within 12-24 hours, and by group 1 participants at 24-48 hours. One week after the administration of antenatal corticosteroids, hyperglycemia persisted in 20 (35.71%) of the 56 participants in group 1, of which six (30%) participants required insulin therapy. On the other hand, 18 (48.64%) participants from group 2 required additional insulin therapy after a week of administration of steroids when compared to pre-steroid administration status.

Conclusions

The findings of this study demonstrate that antenatal betamethasone therapy resulted in worsening hyperglycemia in most pregnant women, regardless of pre-existing glycemic status. These findings highlight the need for close monitoring of blood glucose levels and potential adjustments to medication regimens following antenatal betamethasone administration, irrespective of the pre-existing glycemic status.

## Introduction

Antenatal corticosteroid therapy is commonly administered between 24 and 34 weeks of gestation in cases of threatened preterm labor and is known to significantly reduce fetal complications, such as hyaline membrane disease, intraventricular hemorrhage, and necrotizing enterocolitis, thereby reducing the need for prolonged admission to neonatal care unit and improving the overall survival of the neonate [1]. Despite these benefits, antenatal corticosteroids are known to have several side effects, including worsening blood glucose control in pregnant women with and without pre-existing diabetes mellitus (DM) [2-4]. The ubiquitous presence of steroid receptors throughout the maternal and fetal bodies results in several side effects [2]. The mechanism of corticosteroid-induced hyperglycemia is based on increased gluconeogenesis in the liver, elevated glucagon levels, decreased peripheral utilization of glucose, reduced insulin synthesis, and increased insulin resistance in pregnancy [5]. Normal pregnancy is characterized by relative insulin resistance, which peaks at 24-28 weeks of gestation. The synergistic combination of the effects of pregnancy-induced insulin resistance and those of corticosteroids on blood glucose control can result in a state of transient hyperglycemia and significant derangement of glucose homeostasis. This necessitates treatment following antenatal corticosteroid therapy in pregnant women with or without pre-existing DM [6]. Transient hyperglycemia is often responsible for fetal adverse effects, including neonatal hypoglycemia and hyperbilirubinemia, reduction in respiratory and body movements, and alteration in fetal heart rate (FHR) demonstrated as abnormal FHR tracings on the non-stress test indicative of fetal distress, often resulting in unnecessary instrumental or premature delivery [7-9].

In our study, 93 pregnant women requiring antenatal corticosteroid therapy were divided into two groups, group 1 comprising euglycemic participants and group 2 comprising participants with diabetes. Blood glucose was monitored for three days after administration of the corticosteroid therapy. One week later, participants from group 1 were reassessed for persistent hyperglycemia. Most participants, regardless of their pre-existing diabetes status, developed hyperglycemia. Notably, 35.71% of the group 1 participants had persistent hyperglycemia a week after administration of antenatal steroids, with higher pregnancy-associated weight gain. Additionally, participants from group 2 demonstrated increased insulin requirements.

The dearth of information about the effect of antenatal corticosteroids on maternal glycemic control and the possibility of euglycemic pregnant women developing persistent hyperglycemia after antenatal corticosteroid therapy mandates various modalities of glycemic control therapy. Given the paucity of literature, and particularly the absence of Indian studies in this context, our study contributes significantly by investigating the effect of antenatal corticosteroids on maternal hyperglycemia in pregnant women with and without diabetes and highlights the potential for persistent hyperglycemia developing even in euglycemic women after antenatal corticosteroid therapy. This finding emphasizes the need for close monitoring and developing optimal glycemic management strategies.

## Materials and methods

This single-center, prospective, observational study approved by the institutional ethics committee (NHH/AEC-CL-2018-245) enrolled women with singleton pregnancies at a gestation age of 32-37 weeks warranting antenatal corticosteroid therapy (standard of care). The minimum required sample size was calculated to be 81 participants based on a previous study [4]. Individuals demonstrating a 30% increase in glucose levels after administration of corticosteroids (precision = 10%; confidence level = 95%) were considered for inclusion in the study. Using convenience sampling, we enrolled 93 consecutive participants who came to the Department of Obstetrics & Gynecology at the Mazumdar Shaw Medical and Cancer Center, Narayana Hrudayalaya Ltd., Bangalore from February 2018 to December 2018. Individuals with underlying infections, those using tocolytics (beta-sympathomimetic), and those unwilling to continue regular monitoring and follow-ups were excluded from the study. Written informed consent was obtained from all the participants enrolled in the study.

The study participants were divided into two groups: group 1 comprised pregnant women who were euglycemic during the 75 g oral glucose tolerance test done at the gestational age of 24-28 weeks (n = 56) and group 2 who had DM (n = 37) (pre-existing DM (n = 7) or gestational diabetes mellitus (GDM) (n = 30)). All participants were administered two doses of intramuscular betamethasone 12 mg injection, 24 hours apart [10-12] immediately upon being prescribed antenatal corticosteroid therapy. Following the therapy, we monitored the capillary blood glucose levels using a glucometer (Contour TS, Bayer). Six readings were recorded daily for three consecutive days, including three pre-meal (fasting, pre-lunch, pre-dinner) glucose levels and three one-hour post-meal glucose levels for participants in group 2. On the other hand, one fasting glucose level and one one-hour post-breakfast glucose level were recorded daily for three consecutive days for participants in group 1.

Correction insulin doses were administered based on the capillary blood glucose level readings. Blood glucose levels ≥140 mg/dL were considered significant hyperglycemia [3,13,14], while blood glucose levels ≥160 mg/dL were considered severe hyperglycemia [15,16]. Observations related to the initiation and modifications of the diabetes management regimen were noted. Based on data suggesting that the glycemic effect of betamethasone begins about 6-12 hours after the first dose and lasts up to 5-7 days [10-12], the glycemic status of hyperglycemic participants from group 1 was reassessed a week after corticosteroid therapy. Fasting plasma glucose levels of ≥95 mg/dL and one-hour post-meal glucose levels ≥140 mg/dL were considered abnormal and indicative of persistent hyperglycemia [17].

Microsoft Excel and SPSS (IBM Corp., Armonk, NY, USA) were used for descriptive and inferential statistical analysis of the data. P-values <0.05 were considered statistically significant.

## Results

The participants in group 1 were significantly younger (27.34 ± 4.25 years) compared to group 2 (31.65 ± 4.34 years) (p < 0.001). However, gestational age (33.27 ± 1.39 weeks in group 1 vs. 33.27 ± 1.38 weeks in group 2) and mean body mass index (BMI) (23.55 ± 4.25 kg/m^2^ in group 1 vs. 24.86 ± 3.92 kg/m^2^ in group 2) were not significantly different between the groups (Table 1).

**Table 1:** Baseline characteristic features of study participants. BMI = body mass index; WBC = white blood cell; DM = diabetes mellitus

Parameters	Group 1 (n = 56)	Group 2 (n = 37)	P-value
	Mean ± SD	Mean ± SD	
Age (years)	27.34 ± 4.25	31.65 ± 4.34	<0.001
Gestational age (weeks)	33.27 ± 1.39	33.27 ± 1.38	0.99
BMI (kg/m^2^)	23.55 ± 4.25	24.86 ± 3.92	0.14
Gravida (n)	1.89 ± 1.11	2.08 ± 1.19	0.44
WBC (cells/mm^3^)	6,553.85 ± 1,559.59	6,582.43 ± 1,331.02	0.48
Family history of DM (%)	19.64	40.54	0.28

Threatened preterm labor (TPTL) (47.3%), pre-existing DM (17.2%), gestational hypertension (8.6%), intrauterine growth retardation (6.5%), twins (2.2%), decreased fetal movements (6.5%), antepartum hemorrhage (2.2%), oligohydramnios (2.2%), cholestasis (2.2%), in vitro fertilization (2.2%), fibroid (1.1%), bad obstetric history (1.1%), and Rh isoimmunization (1.1%) were common indications for antenatal steroid therapy. Of these, TPTL was the most common indication.

Before the administration of steroids, participants in group 2 comprised 17 participants who were being treated with insulin, 15 participants being managed by medical nutrition therapy (MNT), and five participants who were treated with oral anti-diabetic medication (OAD) in the form of metformin in addition to MNT (Table 2).

**Table 2:** Treatment modality before and after the administration of steroids in group 2 participants. MNT = medical nutritional therapy; OAD = oral anti-diabetic drug

Treatment modality	
Treatment modality before the administration of steroids	MNT = 15; OAD = 5; insulin = 17
Change of treatment modality after the administration of the steroids	MNT to insulin = 13; OAD to OAD + insulin = 5; continued on MNT = 2

**Figure 1:**
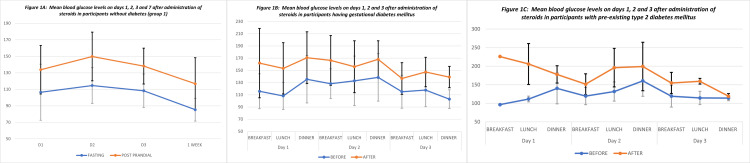
Mean blood glucose levels on days one, two, three, and seven after the administration of steroids in the study participants.

The time of occurrence of significant hyperglycemia requiring corrective insulin doses was earliest in those with pre-existing DM (6 hours), followed by those with GDM (12-24 hours), and, finally, in the euglycemic participants (24-48 hours) (Figures 1A-1C).

Capillary blood glucose levels of ≥140 mg/dL, considered significant hyperglycemia, were seen in 40 (71.4%) participants in group 1 and 34 (91.9%) participants in group 2 (Table 3).

**Table 3:** Comparison of significant hyperglycemia among participants with and without diabetes after the administration of steroids. GRBS = glucometer random blood sugar

Degree of hyperglycemia	Group 1 (n = 56); n (%)	Group 2 (n = 37); n (%)	Total (n = 93); n (%)	P-value
Significant hyperglycemia (GRBS >140 mg/dL)	40 (71.4)	34 (91.9)	74 (79.6)	0.017
Hyperglycemia ( GRBS: ≤140 mg/dL)	16 (28.6)	3 (8.1)	19 (20.4)	

Of the 93 participants, 55 (59.14%) developed severe hyperglycemia, with 24 (42.9%) participants from group 1 and 31 (83.8%) participants from group 2 having capillary glucose levels ≥160 mg/dL (Table 4).

**Table 4:** Comparison of severe hyperglycemia among participants with and without diabetes after the administration of steroids. GRBS = glucometer random blood sugar

Degree of hyperglycemia	Group 1 (n = 56); n (%)	Group 2 (n = 37); n (%)	Total (n = 93); n (%)	P-value
Severe hyperglycemia (GRBS >160 mg/dL)	24 (42.9)	31 (83.8)	55 (59.14)	<0.001
Hyperglycemia (GRBS: ≤160 mg/dL)	32 (57.9)	6 (16.2)	38 (40.9)	

In comparison to baseline insulin requirement before the administration of antenatal steroids, there was a significant increase in the insulin dose for the initial three days following steroid administration in 52.6% of those with GDM compared to 65.86% of those with pre-existing DM (Table 5).

**Table 5:** Glycemic status in participants from groups 1 and 2 (GDM and pre-existing DM) before, during, and one week after antenatal steroid treatment. DM = diabetes mellitus; GDM = gestational diabetes mellitus

Group	Insulin dose period	Count	Mean (average insulin dose/ day)	SD	F	P-value
Group 1	Pre-steroids	56	-	-	6.81	0.041
	During steroid administration	56	4.69	5.69		
	After 1 week	56	2.00	2.83		
GDM	Pre-steroids	30	12.34	20.86	27.65	<0.001
	During steroid administration	30	23.50	23.10		
	After 1 week	30	17.92	21.54		
Pre-existing DM	Pre-steroids	7	33.50	30.21	7.81	0.025
	During steroid administration	7	50.86	22.12		
	After 1 week	7	34.83	29.88		

One week after corticosteroid administration, 20 (35.71%) of the 56 participants in group 1 developed persistent hyperglycemia, requiring interventions for glycemic control (Table 6). These interventions included MNT, OAD, and insulin given to 27, 1, and 26 participants, respectively.

**Table 6:** Persistent hyperglycemia after one week in group 1 participants.

Persistent hyperglycemia after one week in group 1 participants	Number (N)	Percentage (%)	P-value
Present	20	35.7	<0.001
Absent	36	64.3	

The mean weight gain during pregnancy was significantly higher (11.01 ± 2.99 kg) in those with persistent hyperglycemia compared to those who remained euglycemic in group 1 (9.10 ± 2.83 kg) (p = 0.025). Although age and pre-pregnancy BMI were numerically higher in the persistent hyperglycemia group, they were not statistically significant (Table 7).

**Table 7:** Comparing participants from group 1 who remained euglycemic on day seven after antenatal steroid therapy with those who developed persistent hyperglycemia. BMI = body mass index; MNT = medical nutritional therapy; OAD = oral anti-diabetic drug

Parameters	Persistent hyperglycemia (mean ± SD)	Euglycemia (mean ± SD)	P-value
Age (years)	28.6 ± 4.03	26.69 ± 4.18	0.115
Gestational age (weeks)	33.02 ± 1.2	33.40 ± 1.3	0.310
Gravida (count)	1.77 ± 1.1	1.88 ± 1.11	0.73
Pre-pregnancy BMI (kg/m^2^)	24.79 ± 5.21	23.03 ± 3.65	0.155
Weight gain in pregnancy (kg)	11.01 ± 2.9	9.10 ± 2.83	0.025
Therapy required	MNT = 12	-	NA
	MNT + OAD = 2	-	
	Combination insulin = 6		

After a week of antenatal corticosteroids, 26 (46.43%) participants from group 1 required insulin therapy for glycemic control. On the other hand, 35 (94.5%) of the participants from group 2 required insulin therapy compared to 17 (45.95%) participants who were on insulin therapy before steroid administration (Table 2).

## Discussion

Our study noted that antenatal betamethasone therapy resulted in hyperglycemia in a majority of the pregnant participants regardless of the baseline glycemic status. Persistent hyperglycemia was noted in 35.71% of those with previous normal glucose tolerance, and pregnancy weight gain was significantly higher in those who developed persistent hyperglycemia. In those with pre-existing diabetes, there was a 52.6%-65.86% increase in insulin dose requirement during steroid therapy, and an additional 48.64% required insulin after steroid therapy.

The timeline for developing antenatal steroid therapy-related hyperglycemia, with glucose levels peaking later in group 1 compared to group 2, is concordant with observations from previous studies [4,10]. Studies have also noted differences in the time taken to develop maximum hyperglycemia, likely due to variations in methodology, frequency of glucose monitoring, monitoring methods used, and insulin correction protocols used.

The progressively increasing insulin requirements seen in study participants were similar to findings from previous studies [3,4,12,18]. Important risk factors for this increased insulin requirement include obesity, family history of DM, and advanced age. We assessed clinically important hyperglycemia based on two glycemic endpoints, i.e., capillary blood glucose ≥140 mg/dL and ≥160 mg/dL. The ≥140 mg/dL cut-off was considered based on the international guidelines recommending that postprandial blood glucose levels should remain below this value in pregnant women with diabetes [13,14,17]. The ≥160 mg/dL level was based on prior literature linking it to fetal acid-base abnormalities [15,16]. The proportion of participants developing hyperglycemia ≥140 mg/dL after antenatal steroid administration in our study (72% in group 1, 92% in group 2) is similar to other studies reporting 40%-90% rates [3,12,15,18]. This suggests that antenatal corticosteroids cause significant maternal hyperglycemia requiring insulin therapy to prevent adverse effects such as fetal metabolic acidosis [15,16].

Notably, around one-third of group 1 participants developed persistent hyperglycemia one week after steroid therapy. Advanced age (40%), obesity (35%), and family history of DM (20%) emerged as significant risk factors for persistent hyperglycemia through the findings of our study. To our knowledge, there is no prior data on euglycemic pregnant women developing persistent hyperglycemia after antenatal steroid therapy. Mean pregnancy-related weight gain was significantly higher, while age and BMI trended higher (although not statistically significant) in those with persistent hyperglycemia compared to those who remained euglycemic. However, the detection of these differences was beyond the scope of our study.

Our study has several strengths, including being the first Indian study to investigate the effect of antenatal betamethasone therapy on glycemic control in pregnant women with and without DM. The inclusion of participants with and without diabetes enabled better characterization of the effect of antenatal steroid therapy on maternal glycemia. We also demonstrated proof-of-concept for persistent hyperglycemia occurring in euglycemic participants a week after antenatal corticosteroid therapy.

However, there are certain limitations to our study, including the lack of continuous glucose monitoring, which could have captured observations related to the glycemic status more comprehensively. Additionally, we did not assess maternal glycemic status at delivery, fetal cord blood pH, neonatal well-being, or neonatal glucose levels. These parameters could have provided a more holistic understanding of the relationship between maternal hyperglycemia and fetal outcomes.

## Conclusions

To our knowledge, our study is the first in the Indian context to investigate persistent hyperglycemia in euglycemic pregnant women receiving antenatal corticosteroids. The findings of our study indicate the need for close follow-up and intensive monitoring protocols after antenatal steroid therapy even for euglycemic pregnant women, especially for those with advanced age, obesity, or family history of diabetes. This will aid in early diagnosis and effective management of persistent hyperglycemia, potentially reducing maternal and fetal morbidity.
